# Zika Virus Infection of Sertoli Cells Alters Protein Expression Involved in Activated Immune and Antiviral Response Pathways, Carbohydrate Metabolism and Cardiovascular Disease

**DOI:** 10.3390/v14020377

**Published:** 2022-02-11

**Authors:** Mahamud-ur Rashid, Ying Lao, Victor Spicer, Kevin M. Coombs

**Affiliations:** 1 Department of Medical Microbiology and Infectious Diseases, The University of Manitoba, Room 543 Basic Medical Sciences Building, 745 Bannatyne Avenue, Winnipeg, MB R3E 0J9, Canada; kevin.coombs@umanitoba.ca; 2 Manitoba Centre for Proteomics & Systems Biology, Room 799, 715 McDermot Avenue, Winnipeg, MB R3E 3P4, Canada; ying.lao@umanitoba.ca (Y.L.); victor.spicer@umanitoba.ca (V.S.); 3 Children’s Hospital Research Institute of Manitoba, Room 513, John Buhler Research Centre, 715 McDermot Avenue, Winnipeg, MB R3E 3P4, Canada

**Keywords:** Zika virus, Sertoli cells, sexual transmission, persistence in semen, immune response, glycolysis, carbohydrate metabolism, cardiovascular disease

## Abstract

Zika virus (ZIKV), a re-emerging virus, causes congenital brain abnormalities and Guillain–Barré syndrome. It is mainly transmitted by Aedes mosquitoes, but infections are also linked to sexual transmissions. Infectious ZIKV has been isolated, and viral RNA has been detected in semen over a year after the onset of initial symptoms, but the mode of long-term persistence is not yet understood. ZIKV can proliferate in human Sertoli cells (HSerC) for several weeks in vitro, suggesting that it might be a reservoir for persistent ZIKV infection. This study determined proteomic changes in HSerC during ZIKV infections by TMT-mass spectrometry analysis. Levels of 4416 unique Sertoli cell proteins were significantly altered at 3, 5, and 7 days after ZIKV infection. The significantly altered proteins include enzymes, transcription regulators, transporters, kinases, peptidases, transmembrane receptors, cytokines, ion channels, and growth factors. Many of these proteins are involved in pathways associated with antiviral response, antigen presentation, and immune cell activation. Several immune response pathway proteins were significantly activated during infection, e.g., interferon signaling, T cell receptor signaling, IL-8 signaling, and Th1 signaling. The altered protein levels were linked to predicted activation of immune response in HSerC, which was predicted to suppress ZIKV infection. ZIKV infection also affected the levels of critical regulators of gluconeogenesis and glycolysis pathways such as phosphoglycerate mutase, phosphoglycerate kinase, and enolase. Interestingly, many significantly altered proteins were associated with cardiac hypertrophy, which may induce heart failure in infected patients. In summary, our research contributes to a better understanding of ZIKV replication dynamics and infection in Sertoli cells.

## 1. Introduction

Zika virus (ZIKV) belongs to the family Flaviviridae, and is a single-stranded positive-sense RNA virus. Other members of the family include Japanese encephalitis virus (JEV), Yellow fever virus (YFV), West Nile virus (WNV) and Dengue virus (DENV). ZIKV was first discovered in Rhesus monkeys in Uganda’s Zika rainforest in 1947 [1].

However, the virus remained undiagnosed for a long time due to the disease’s non-specific flu-like symptoms and a lack of diagnostic screening [2,3]. In 2007, ZIKV re-emerged in the Pacific islands, spreading to over 80 countries/territories, including Latin America, the United States, and Southeast Asia [4,5,6,7,8]. The virus has been linked to microcephaly in babies [9] and Guillain–Barré syndrome (GBS) in adults [10,11]. Therefore, the World Health Organization (WHO) considers ZIKV to be a threat to global health, and declared a global health emergency in 2016 [12].

In endemic regions, ZIKV is mainly transmitted by mosquito bites (*Aedes aegypti* and *A. albopictus*) [13]. However, the virus can persist in the male reproductive tract and contribute to sexual transmission [14,15,16]. After the initial onset of symptoms, ZIKV RNA and infectious virus particles were detected in semen from infected males after 414 days [17] and 69 days [18], respectively. Sexual transmission of ZIKV has been reported in 14 countries so far [19]. ZIKV was found in murine testis and monkey models after inoculation, causing severe testicular damage [20,21,22,23]. Unlike the animal models, ZIKV infection does not cause any noticeable impact on human testis morphology. However, persistent ZIKV infection in the male genital tract may impact testicular hormone levels and sperm quality [24,25,26]. These findings indicate that, like many other viruses [27], ZIKV exploits the immune-privileged environment of the testis, which lacks an adaptive immune system [28], and hides for a prolonged time. However, the cellular reservoir of ZIKV testicular persistence is not clearly understood.

Sertoli, Leydig, and germ cells are the most prevalent cells found in human testes [29]. Sertoli cells constitute the major part of the testicular environment and play a critical role in the development of spermatogonial stem cells into mature sperm [30,31]. The testis–blood barrier, which protects the male reproductive system from pathogens, is also established by Sertoli cells [32]. Another major cell type in the testes is the Leydig cell, which secretes the male sex hormone testosterone and is essential for the development of male reproductive organs and characteristics [33]. In mouse testis, Sertoli cells are the most susceptible to ZIKV infection [34]. A previous study has demonstrated that ZIKV can cross the blood–testis barrier and efficiently infect Sertoli cells [35]. Moreover, the virus not only can infect Sertoli cells, but can persist for several days/weeks without any observable cytopathic impact [34,36,37]. 

We previously used an aptamer-based assay and demonstrated that ZIKV infection caused alterations in the levels of proteins involved in spermatogenesis in HSerC [36]. In order to better understand the mechanism(s) of viral persistence and its impact on the male genital tract, we extended the previous study by using a complementary tandem mass tag (TMT)-based 2D LC/MS/MS mass spectrometry-based approach to investigate Sertoli cell proteomic alterations after ZIKV infection. We measured ~8000 proteins across three time points and identified approximately 4400 Sertoli cell proteins significantly affected by ZIKV. The possible significance of these proteins in cellular processes, signaling pathways, and disease pathogenesis were investigated through bioinformatics analyses.

## 2. Materials and Methods

### 2.1. Cells

Primary Sertoli cells (HSerC) isolated from human testis were purchased from ScienCell Research Laboratories, CA, USA (Catalog #4520). The cells were cultured at 37 °C in 5% CO_2_ in poly-l-lysine-(Sigma−Aldrich, ON, Canada, Cat.# P4707) coated culture plates in Sertoli Cell Medium (ScienCell Research Laboratories, CA, USA, Cat. #4521). Following the company’s recommendation, Sertoli cells were trypsinized to detach them from the culture surface and passaged every 2–3 days for maintenance. All experiments were conducted using the cells that had reached passage 7.

### 2.2. Virus

The Zika virus strain used in this study (ZIKV/Homo sapiens/PRI/PRVABC59/2015) was donated by Dr. David Safronetz, Chief of Special Pathogens, National Microbiology Laboratory, Public Health Agency of Canada. For future use, the ZIKV strain was expanded in Vero cells (ATCC, Manassas, VA, USA, Cat. #CCL-81) and kept at −80 °C in 10% FBS (Thermo Fisher Scientific, Waltham, CA, USA, Cat #A4766801). The stock virus was titered in Vero cells by plaque assay. 

### 2.3. Infection

HSerC were grown in 75 cm^2^ cell culture flasks treated with poly-l-lysine. At 70% confluency, cells were infected with ZIKV at a multiplicity of infection (MOI) of 3 plaque-forming units (PFU) per cell. Culture plates were rocked every 10–15 min for 2 h in a 37 °C incubator (Thermo electron corporation, Waltham, MA, USA) to allow the virus to attach to cell surfaces, and cells were overlaid with Sertoli cell media containing 2.5% FBS. At 3, 5, and 7 days after infection, ZIKV-infected and mock, non-infected cells were collected. All experiments were done in three biological replicates. 

### 2.4. Protein Extraction and Quantification

The Sertoli cells infected with ZIKV and time-matched mock-treated cells were scraped from the culture plates after 3, 5 and 7 days of infection. Centrifugation at 600× *g* for 8 min pelleted the cells, which were then washed three times with sterile ice-cold PBS(Thermo Fisher Scientific, Waltham, CA, USA). The pelleted cells were then lysed in 4% SDS in 100 mM HEPES buffer pH 8.5 by sonication. Centrifugation at 14,000× *g* for 10 min at 4 °C was used to remove insoluble cellular components. Bradford Protein Assay was used to determine the protein concentrations in the supernatants (Bio-Rad, Hercules, CA, USA, Cat. 5000001).

### 2.5. Immunoblotting

The protein concentrations in the cell lysates were measured using the Bradford Protein Assay, and 20 µg of protein were resolved in 10% SDS-PAGE gels and transferred to 0.2 µm nitrocellulose membranes. Anti-PSMA2 (Cell Signaling, Danvers, MA, USA, Cat. 2455), anti-ZIKV NS1 (BioFront Technologies, Tallahassee, FL, USA, Cat. BF-1225-06), anti-ZIKV NS3 (Genetex, Irvine, CA, USA, Cat No. GTX133309), anti-ZIKV Env (Genetex, Cat No. GTX133314), anti-STAT1 (Cell Signaling, Cat. 9176S), and anti-Beta-Actin (Cell Signaling, Cat. 3700S) antibodies were used to detect protein targets. Anti-rabbit (Cell Signaling, Cat. #7074) or anti-mouse (Cell Signaling, Cat. #7076) secondary antibody was used to identify the primary antibody conjugates to the targeted proteins. After overlaying with ECL reagents, protein bands were photographed with an Amersham Imager 680 (Gelifesciences, MA, USA). To quantify band intensities, Image J version. 1.53e (NIH, Bethesda, MD, USA) was used, and Graphpad Prism version 6.0. (La Jolla, CA, USA) was used to visualize them graphically.

### 2.6. Tandem Mass Tags (TMT) Mass Spectrometry Analyses and Protein Quantification

To determine the impact of ZIKV infection on the cellular proteome, a total of 18 protein samples were collected from ZIKV- and mock-infected Sertoli cells at 3, 5 and 7 days post-infection (dpi). Proteins were digested into peptides by the SP3 (single-pot solid-phase-enhanced sample preparation) methods as described elsewhere [38,39]. In summary, peptides were eluted after digestion of the proteins with trypsin for 14 h at 37 °C. Six-plex TMT labeling was performed for mock and infected samples of the same time points following the manufacturer’s (Thermo Fisher Scientific, Waltham, CA, USA) instructions. An equal amount of six TMT labeled samples were mixed together and 2D LC/MS/MS was performed using an Orbitrap Q Exactive HF-X instrument [40] (Thermo Fisher Scientific, Bremen, Germany). For identification of the peptide/proteins, ZIKV (Thai strain) and human (Uniprot 2016) databases were used as references. The intensities of TMT6 peptide level reporter tags were averaged over a ±0.1 Da window and corrected for isotopic overlap between channels using the batch-specific correction matrix provided. The sum of peptide level TMT6 reporter tag intensities for each protein was transformed into a log_2_ scale for easier differential analysis. 

### 2.7. Statistical and Bioinformatics Analyses

Initially, alterations in the levels of any individual protein expression were determined by the differences between Log_2_ values (Delta log) of an infected and time-matched mock sample. Then, the delta Log_2_ values were converted to fold-change for each of the proteins. The *p*-value was determined by the Students *t*-test (2 tails) and Z-score analyses based on the protein expression difference of all three replicates. *p*-value < 0.05 and Z-score values of ≥.96 σ and ≤−1.96 σ were considered significant as described before [41]. The lists of significantly altered proteins were uploaded into Ingenuity Pathway Analysis (IPA) software, and core analysis was done with a cut-off value *p*-value < 0.05 and fold change above 1.5 or below −1.5. The IPA core analysis predicted the top affected canonical pathways, bio-functions, interconnecting networks and upstream molecules based on these protein level changes. The Western blot image band intensities were quantified using Image J version1.53e software (NIH, Bethesda, MD, USA), and statistical analyses were performed by one-way or two-way ANOVA (*p*-values < 0.05) in GraphPad Prism version 6.0. MORPHEUS (Broad Institute, Cambridge, MA, USA), a free internet software program, was used to generate the heatmaps.

## 3. Results

### 3.1. Infectivity of ZIKV and Its Cytopathic Effect in Primary HSerC 

**Figure 1:**
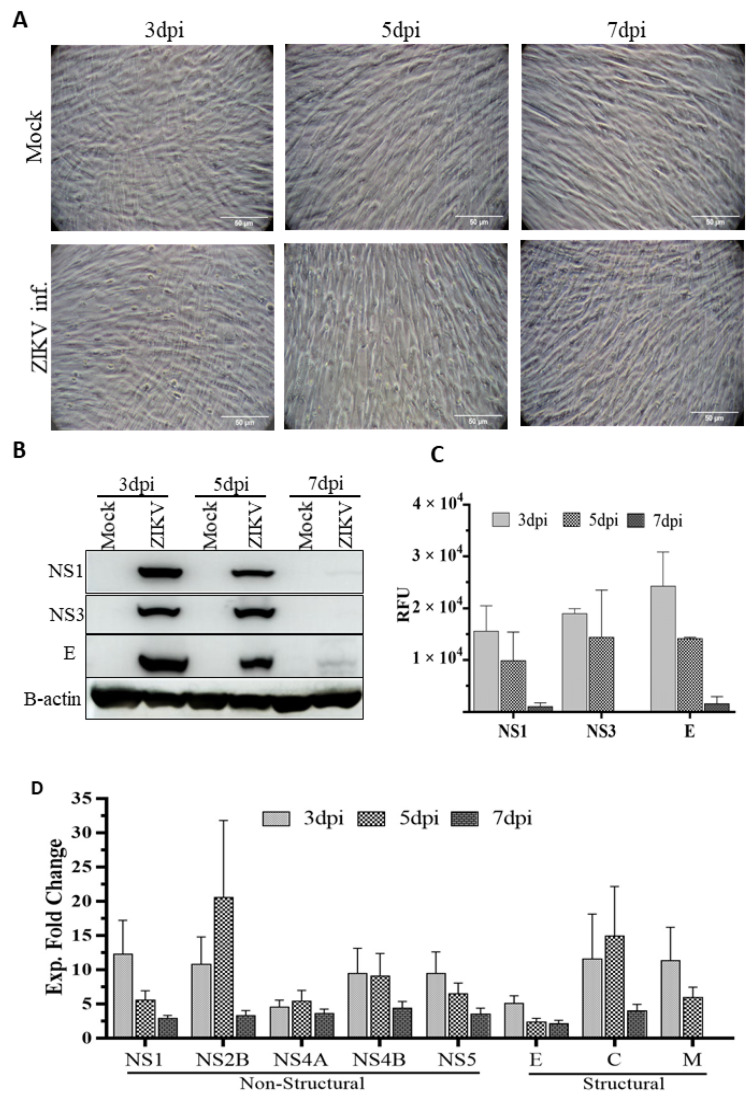
Cytopathic impact of ZIKV infection on HSerC and viral protein expression. HSerC were infected with ZIKV an MOI of 3. (**A**) Cytopathic impact of ZIKV infection was observed at 3, 5 and 7 days post infection (dpi) under bright−field microscopy at 200× magnification. Scale bar is 50 μm. (**B**) Viral protein expression was determined by Western blot using ZIKV-NS1, NS3 and ENV monoclonal antibodies at 3, 5 and 7 dpi. (**C**) Quantitative expression of ZIKV viral proteins determined by densitometry analysis of Western blot images using Image J and normalized to B-actin expression. (**D**) ZIKV proteins expression detected after 3, 5 and 7 dpi by mass spectrometry. Log2 expressions of ZIKV proteins were compared with mock-treated cells and converted to Fold Change (FC) Abbreviations. dpi = Days post infection. Exp = Expression. RFU = Relative fluorescence units.

After infecting HSerC with ZIKV at MOI = 3, viral protein expression levels, which could result from increased expression, lower protein turnover, or a combination of both, and cytopathic effects were monitored at 3, 5 and 7 dpi, ZIKV infection did not induce any cytopathic effects (Figure 1A). However, three viral proteins (NS1, NS3, and E) were expressed at all time points in the infected cells, with maximal levels at 3 dpi; by 5 and 7 dpi, their expressions had dropped significantly (Figure 1B,C). Mass spectrometry analysis of Zika viral protein levels revealed that most of the proteins were at their highest levels at 3 dpi, with the exception of NS2B, NS4A, and C, which peaked at 5 dpi. All ZIKV proteins were expressed to the lowest level at 7 dpi (Figure 1D). In our previous study, we did not observe any significant protein level alterations at the early stage (1 dpi) of replication. However, virus titer peaks at 5 dpi and declines at 7 dpi [36]. Therefore, we selected day 3 (mid), day 5 (peak viral titer) and day 7 (late stage) for subsequent proteomic analyses based on these observations.

### 3.2. The Impact of ZIKV Infection on the HSerC Cellular Proteome

**Figure 2:**
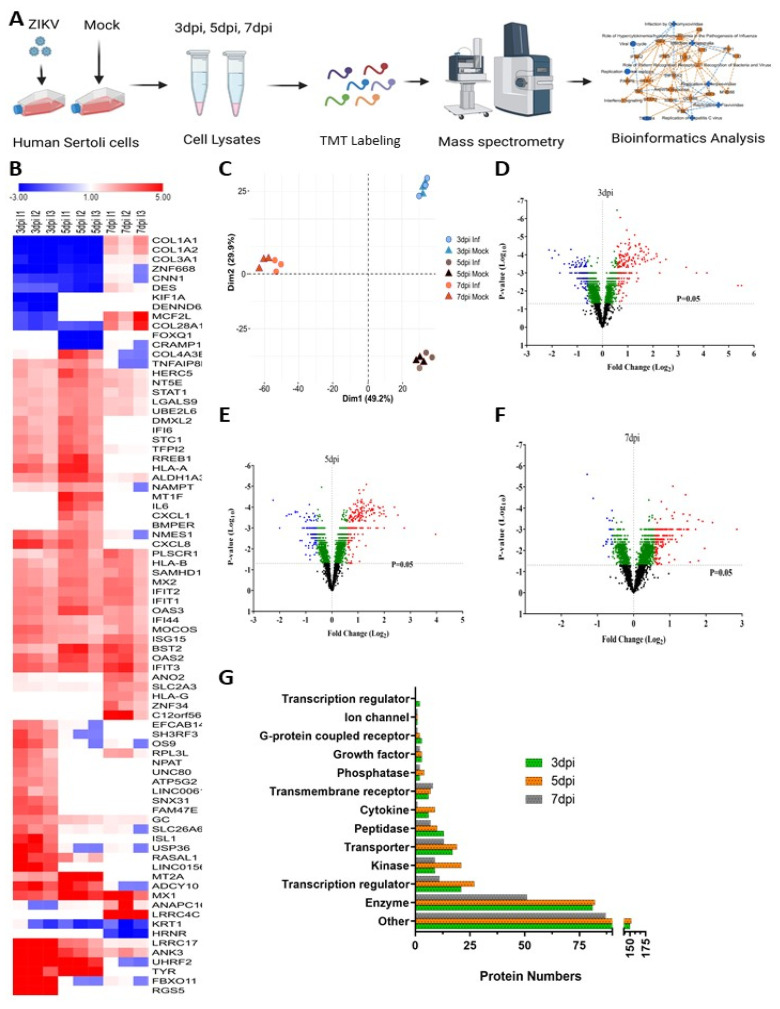
Detection of cellular protein level alterations in ZIKV-infected Sertoli cells by mass spectrometry. (**A**) Schematic flow diagram of the study design. (**B**) Heatmap of most affected proteins (Fold change ≥ 2.5 or ≤−2.5; *p* value < 0.05) by ZIKV infection in Sertoli cells. Red and blue colors indicate up-regulation and down-regulation, respectively. (**C**) PCA plot of proteomic data from mock- and ZIKV-infected cells, from all three replicates. Volcano plots displaying the protein level alterations after ZIKV infection at 3 dpi (**D**), 5 dpi (**E**), and 7 dpi (**F**). Red = significantly up-regulated (FC > 1.5; *p*-value < 0.05), blue = significantly down-regulated (FC < −1.5; *p*-value < 0.05), green = significantly affected (*p*-value < 0.05, but FC < ±1.5), black = not significantly affected (*p*-value > 0.05). (**G**) Classification of significantly altered (Fold change ≥ 1.5 or ≤ −1.5; *p* value < 0.05) protein types at different time points after ZIKV infection. Abbreviations. FC = Fold change; dpi = Days post infection; inf = Infected.

TMT-based mass spectrometry was used to determine the proteomic alterations in protein levels caused by ZIKV infection in HSerC, which detected more than 6000 proteins from each sample (Figure 2A). Among them, 4423 unique protein levels were significantly affected considering all three time points. A total of 2367 (853 up-regulated and 1514 down-regulated), 2363 (1651 up-regulated and 712 down-regulated), and 1782 (1209 up-regulated and 573 down-regulated) proteins were significantly (*p* value < 0.05) affected at 3, 5 and 7 dpi, respectively (Table 1). 

Heatmaps of the most affected proteins (Fold change ≥ 2.5 or ≤ −2.5; *p*-value < 0.05) revealed that certain protein levels were affected at specific time periods while other proteins were significantly affected at all times (Figure 2B). The topmost up-regulated proteins were Regulator of G-protein signaling 5 (RGS5, Fold change (FC): 45.4), F-box only protein 11 (FBXO11, FC: 41.1), E3 ubiquitin-protein ligase UHRF2 (UHRF2, FC: 17.5), Tyrosinase (TYR, FC: 15.6), Ankyrin-3 (ANK3, FC: 13.2) and the down-regulated were Forkhead box protein Q1 (FOXQ1, FC: −4.74), Kinesin-like protein (KIF1A, FC: −4.37), Collagen alpha-1(I) chain (COL1A1, FC: −3.95), Protein cramped-like (CRAMP1, FC: −4.02), and Hornerin (HRNR, FC: −3.93) (Figure 2B, Table 2). Principal component analysis (PCA) also showed that the protein samples were clustered by time point (Figure 2C). 

The volcano plot analysis of the proteins revealed that the majority of the highly affected proteins were down-regulated at 3 dpi (Figure 2D). In contrast, the most significantly up-regulated were at 5 and 7 dpi (Figure 2E,F). The altered proteins belong to different classes of proteins, including enzymes, transcription regulators, kinases, transporters, peptides, cytokines, transmembrane receptors, phosphatases, G protein-coupled receptors, growth factors, ion channels, transcription regulators and others (Figure 2G). We applied +/−1.5-fold fold-change cut-off with *p* < 0.05 for subsequent more complete bioinformatics analysis.

### 3.3. Impact of ZIKV Infection on Cellular Signaling Pathways and Function in HSerC

**Figure 3:**
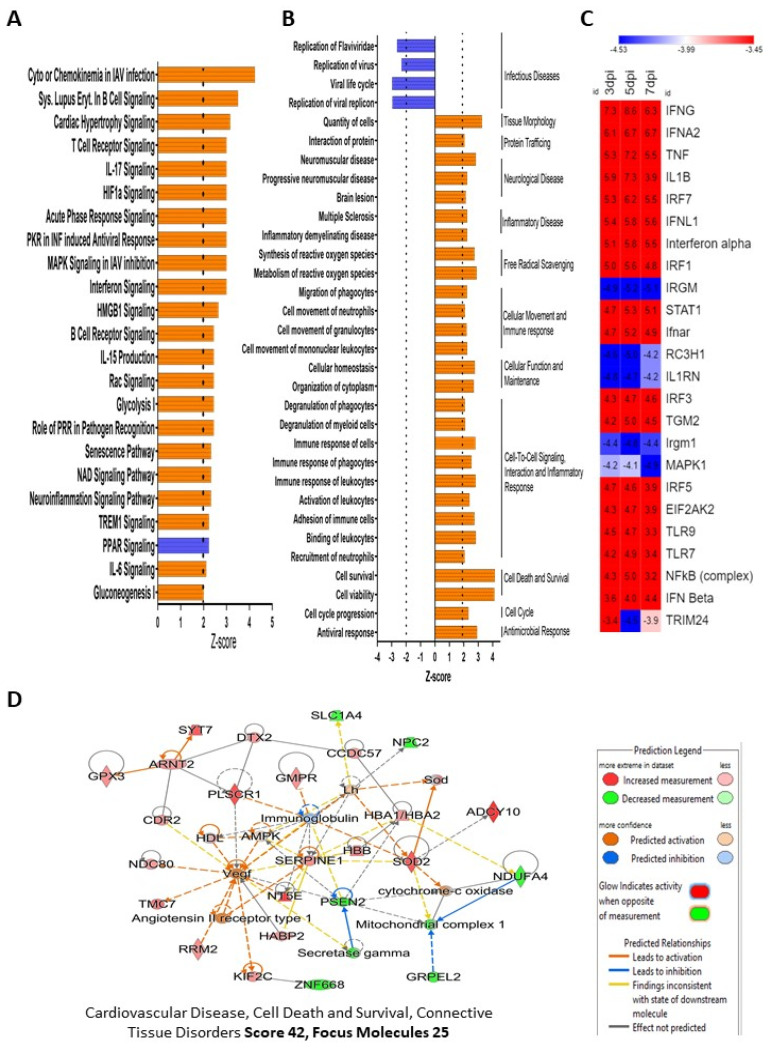
IPA-predicted activation and inhibition of bio-functions, canonical pathways, protein–protein networks, and upstream molecules after ZIKV infection in HSerC. (**A**) Top-most canonical pathways predicted to be significantly activated or inhibited by IPA at 5 days post ZIKV infection. (**B**) Top bio-functions and predicted activation or inhibition Z-scores indicated at 5 dpi. Activation is indicated by a positive Z-score and the inhibited bio-functions are indicated by negative Z-scores. (**C**) The top 25 upstream regulators, predicted to be affected at 3, 5 and 7 dpi after ZIKV infection in HSerC. Red indicates up-regulation, blue indicates down-regulation. Numbers in boxes show the significance of alteration measured by Z-score. (**D**) The most affected protein networks at 5 dpi by ZIKV infection in HSerC. Red and green represent up-regulation and down-regulation, respectively; gray proteins denote that they were recognized in the present study, but not significantly regulated; colorless proteins interact with molecules in the network, but were not identified in our study. Abbreviations. dpi = Days post infection.

All proteins and their measured quantities at 3, 5 and 7 dpi were analyzed by Ingenuity Pathway Analysis (IPA) software to understand predicted impacts of ZIKV infection on cellular signaling pathways, bio-functions, and protein–protein networks. A total of 15, 31 and 13 Sertoli cell canonical pathways were predicted to be significantly affected at 3, 5 and 7 dpi, respectively, after ZIKV infection (Figure 3A, Figure S1A). GP6 signaling pathway was inhibited at 3 dpi, but was activated significantly at 7 dpi. Natural killer cell signaling was significantly down-regulated only at 7 dpi. Seven signaling pathways were predicted to be significantly activated at all three-time points; they are hypercytokinemia/hyperchemokinemia in the pathogenesis of influenza, interferon signaling, Systemic Lupus Erythematosus in B cell signaling pathway, role of PKR in interferon induction and antiviral response, neuroinflammation signaling pathway, role of pattern recognition receptors in recognition of bacteria and viruses, and NAD signaling pathway (Figure 3, Figure S1A). Interestingly, a few canonical pathways were only affected at 5 dpi, e.g., IL-17 signaling, IL-6 signaling, Cardiac hypertrophy signaling (enhanced), HIF1α signaling, Glycolysis I, Gluconeogenesis I, PPAR signaling, HMGB1 signaling, Rac signaling, etc. IPA predicted the altered proteins were significantly (Z score >1.96 or <−1.96) associated with a total of 156 diseases and functions, which were divided into 55 for 3 dpi, 95 for 5 dpi and 63 for 7 dpi (Table S1 Pathways associated with infectious disease were predicted to be significantly inhibited, whereas those associated with antimicrobial response, inflammatory disease, neurological disease, tissue morphology, cell death and survival, and cellular movement were the major disease and cellular function categories predicted to be activated at all three time points (Figure 3B; Figure S2). IPA also predicted a total of 801 unique upstream regulators to be activated/inhibited by ZIKV infection in Sertoli cells at 3 dpi (*n* = 400, activated, *n* = 279; inhibited 121), 5 dpi (*n* = 616, activated, n = 442; inhibited 174), and 7 dpi (*n* = 299, activated, *n* = 206; inhibited 93), which includes cytokines, enzymes, G-protein coupled receptors, growth factors, kinases, microRNAs, peptidases, phosphatase, transcription regulators, transmembrane receptor and transporters (Table S2). The top ten predicted upstream regulators are IFNG, IFNA2, TNF, IL1B, IRF7, IFNL1, Interferon-alpha, IFR1, IRGM and STAT1 (Figure 3C). The functional analysis of the top 15 upstream regulators showed that they are associated with activation of T-lymphocytes, leukocytes, cells, recruitment of leukocytes, antiviral response, and RNA virus replication (Figure S1B). 

IPA also built protein–protein networks of the significantly affected proteins based on their direct or indirect interactions. There were 12, 9, and 10 protein–protein interaction networks (Score > 20, Molecules > 13) predicted by IPA analysis of the altered proteins at 3, 5 and 7 dpi, respectively. The predicted networks were associated with different cellular functions and diseases, including cell morphology, cellular assembly and organization, neurological disease, lipid metabolism, molecular transport, cardiovascular disease, connective tissue disorders, antimicrobial response, cell cycle, and endocrine system development (Table S3). Cancer, connective tissue disorders, organismal injury and abnormalities (Score 38, Focus Molecules 23) were the most affected predicted protein–protein networks at 3 dpi, cardiovascular disease, cell death and survival, and connective tissue disorders (Score 42, Focus Molecules 25) were the most affected at 5 dpi, and antimicrobial response, and infectious diseases, inflammatory response (Score 41, Focus Molecules 22) were predicted to be the most affected at 7 dpi (Figure 3D; Figure S2C,D). 

### 3.4. HSerC Activates Immune Response against ZIKV Infection

**Figure 4:**
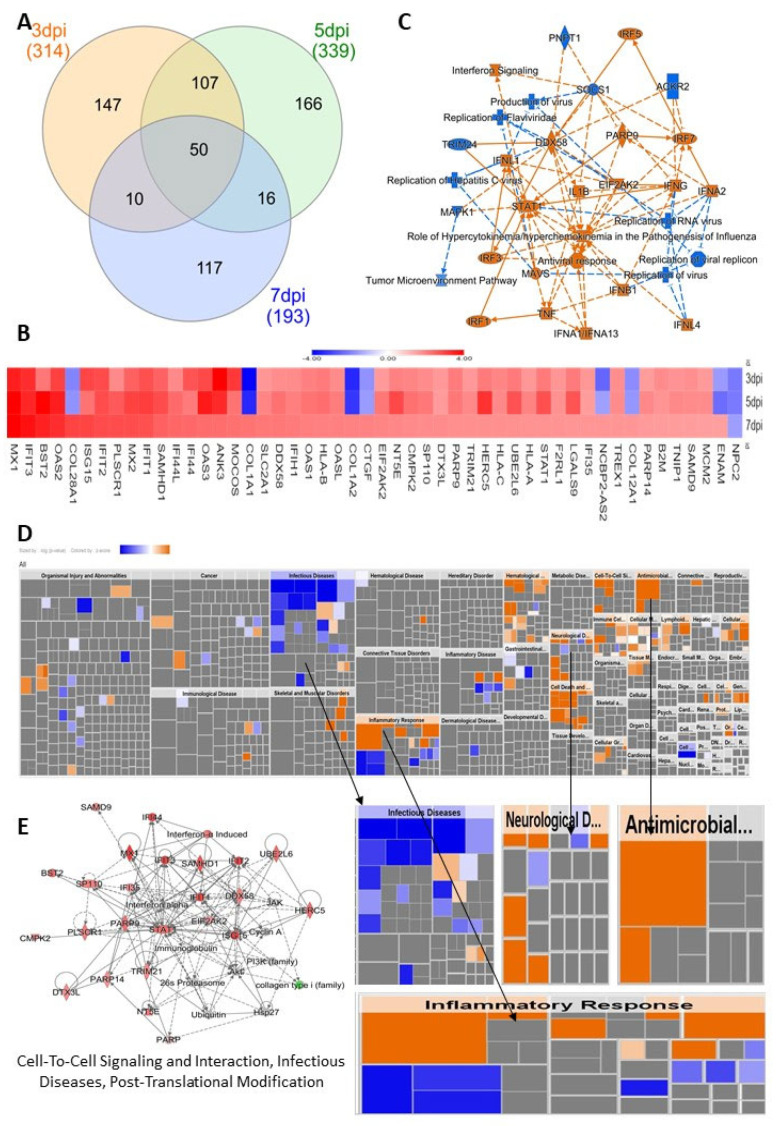
Impact of commonly affected proteins in HSerC by ZIKV on cellular functions and diseases. (**A**) Venn diagram of altered proteins at 3, 5 and 7 dpi. (**B**) Summary of IPA analysis showing the most affected bio-functions, canonical pathways and affected protein networks and their relationship by the commonly affected proteins. (**C**) Heatmap of the 50 commonly affected proteins at 3, 5 and 7 dpi. Red/orange and blue represent up-regulation and down-regulation, respectively. (**D**) Heat map of the disease and biofunctions affected by the commonly affected proteins. (**E**) The protein–protein interaction networks affected by the commonly altered proteins. Red and green represent up-regulation and down-regulation, respectively; gray proteins denote that they were recognized in the present study, but not significantly regulated; colorless proteins interact with molecules in the network, but were not identified in our study. Abbreviations. dpi = Days post infection.

To understand the impact of ZIKV infections across the three time points, we selected the list of 50 commonly affected proteins (Figure 4A,B) and performed IPA core analysis. This analysis showed that the most affected proteins were predicted to be associated with canonical pathways involving replication of flavivirus, replication of RNA virus and interferon signaling pathways (Figure 4C). The infectious disease, inflammatory response, neurological disease, and antimicrobial disease responses were the most affected disease and functions predicted by heatmap analysis of IPA (Figure 4D). The protein–protein network analysis predicted their links with cell−to−cell signaling and interaction, infectious diseases, and post−translational modification (Figure 4E).

**Figure 5:**
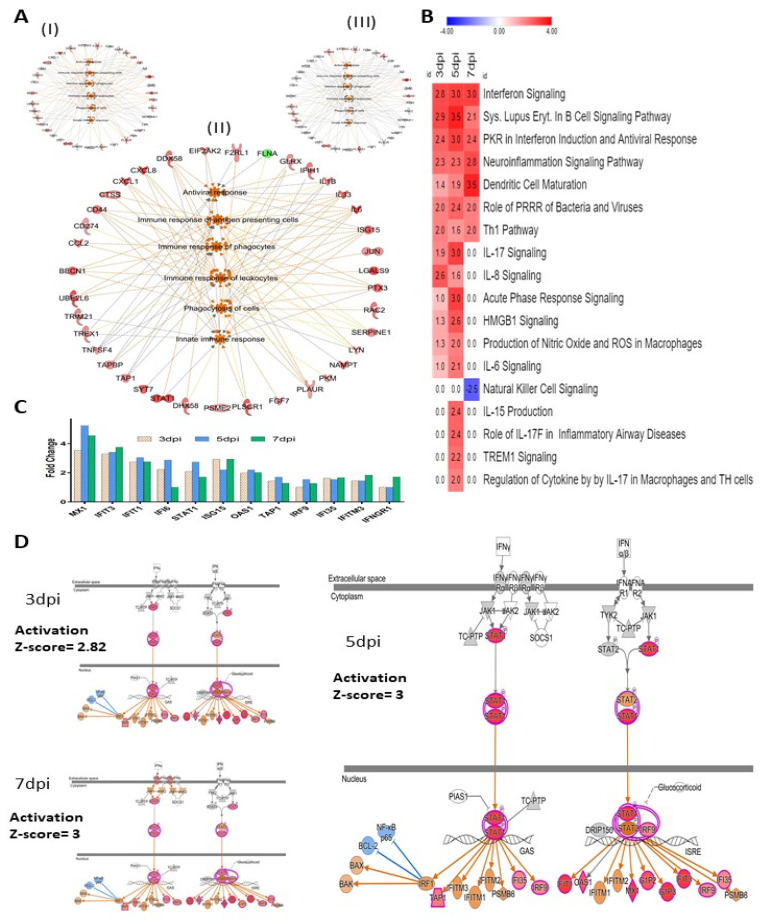
Immune response in HSerC after ZIKV infection. (**A**) Bio-functions related to immune response pathways affected at (I) 3 dpi, (II) 5 dpi, and (III) 7 dpi. (**B**) Canonical pathways associated with immune response pathways affected by ZIKV infection in Sertoli cells. Red and blue represent up-regulation and down-regulation, respectively. The number in each box shows the activation Z-score predicted by IPA. (**C**) Proteins in Interferon signaling pathways affected by ZIKV infection. (**D**) Activation of Interferon signaling pathway by ZIKV infection. Red and green represent up-regulation and down-regulation, respectively; gray proteins denote that they were recognized in the present study, but not significantly regulated; colorless proteins interact with molecules in the network, but were not identified in our study. Abbreviations. dpi = Days post infection.

The immune response of antigen-presenting cells, immune response of phagocytes and leukocytes, activation of antiviral response, phagocytosis of cells and innate immune response also were predicted to be activated at 3, 5 and 7 days post-ZIKV infection (Figure 5A). Several proteins involved in immune response regulating signaling pathways were also significantly stimulated by ZIKV infection, which includes interferon signaling, PKR interferon induction and antiviral response, role of pattern recognition receptor in recognition (PRRR) of bacteria and virus, Th1 pathway, HMGB1, TREM1, IL-17, IL-8, IL-6, IL-15 signaling, etc. (Figure 5B). Across the three time points after ZIKV infection, interferon signaling was one of the most affected signaling pathways in Sertoli cells. ZIKV caused significant alteration of 12 protein levels that are predicted to regulate interferon signaling pathways (Figure 5C). Based on the expression values of these proteins, IPA predicted a significant activation of the pathways at 3 dpi (Z-score 2.82), 5 dpi (Z-score 3.0), and 7 dpi (Z-score 3.0) (Figure 5D). 

**Figure 6:**
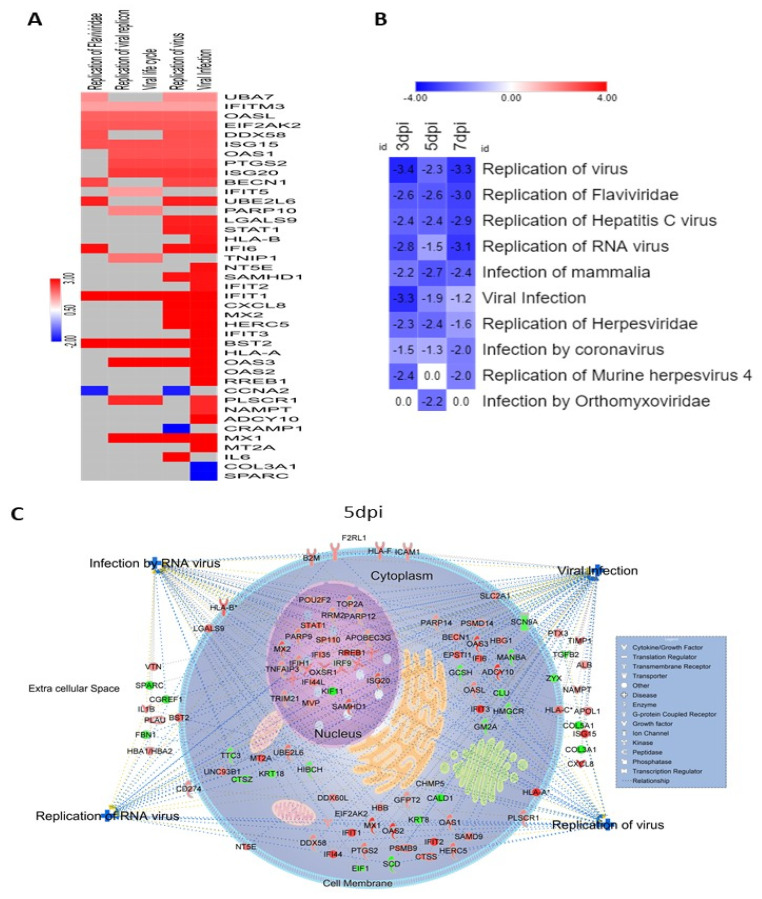
Viral replication was suppressed by the cellular response in HSerC after ZIKV infection. (**A**). Heat map of the proteins involved in virus infection, lifecycle and replication at 5 dpi. Red and blue represent up-regulation and down-regulation of protein expression, respectively. (**B**) Replication of different family of viruses were predicted to be inhibited by the significantly affected proteins at 3, 5 and 7 dpi. The number in the box indicates the Z-score of the representative function or disease. (**C**) Cellular localization of the altered proteins predicted to be involved in the regulation of viral infection and in infection by, and replication of, RNA viruses. Red and green represent up-regulation and down-regulation of protein expression, respectively. Abbreviations. dpi = Days post infection.

We identified several proteins associated with establishment of viral infection and replication that were significantly affected by ZIKV infection in HSerC (Figure 6A). Based on their expressions, IPA predicted that proteins associated with Flaviviridae infection and replication were significantly inhibited (Figure 6B). Interestingly, these analyses also predicted that proteins associated with replication of some other viruses such as Hepatitis C virus, Herpesviridae, murine herpesvirus 4, Orthomyxoviridae, and coronavirus were affected (Figure 6B). These proteins associated with viral infection and replication are predicted to be found in the cytoplasm, nucleus, cell membrane, and extracellular space of Sertoli cells (Figure 6C).

### 3.5. ZIKV Infection Impacts Carbohydrate Metabolism in Sertoli Cells

**Figure 7:**
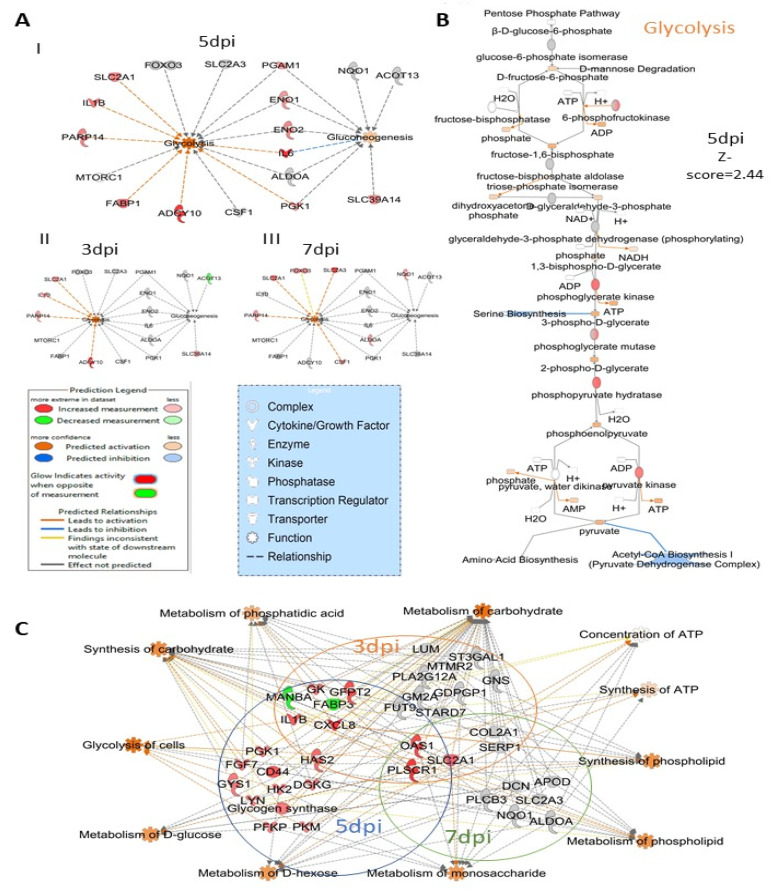
ZIKV infection impacts carbohydrate metabolism in HSerC. (**A**) Proteins and enzymes involved in glycolysis and gluconeogenesis pathways affected by ZIKV infection at I. 5 dpi, II. 3 dpi and III. 7 dpi. IPA predicted the impact of ZIKV infection on (**B**) glycolysis pathway at 5 dpi in Sertoli cells. (**C**) Proteins involved in carbohydrate metabolism and energy production in cells affected by ZIKV infection. Red and green represent up-regulation and down-regulation of protein levels, respectively. Abbreviations. Dpi = Days post infection.

At 5 dpi ZIKV caused significant activation of glycolysis (Z-score = 2.44) and gluconeogenesis (Z-score = 2.0) pathways (Figure 3A). There were 18 Sertoli cell proteins associated with the glycolysis and gluconeogenesis pathway that were significantly affected by ZIKV infection. Eleven of their levels (SLC2A1, IL1B, PRP14, FABP1, ADCY10, PGK1, IL6, ENO2, ENO1, PGAM1 and SL39A14) were altered (Figure 7A) at 5 dpi and predicted to significantly activate (Z score = 2.44) the glycolysis pathway (Figure 7B). ZIKV infection significantly affected 20, 19 and 11 proteins related to carbohydrate metabolism and cellular energy production pathways at 3, 5 and 7 dpi, respectively (Figure 7C; Figure S3). These proteins are associated with the synthesis of ATP and its concentration in cells, metabolism of carbohydrate, phosphatidic acid, d-glucose, d-hexose, monosaccharide, phospholipids and synthesis of carbohydrate and phospholipid. 

### 3.6. ZIKV Infection Significantly Affects Proteins That May Increase Cardiovascular Disease

**Figure 8:**
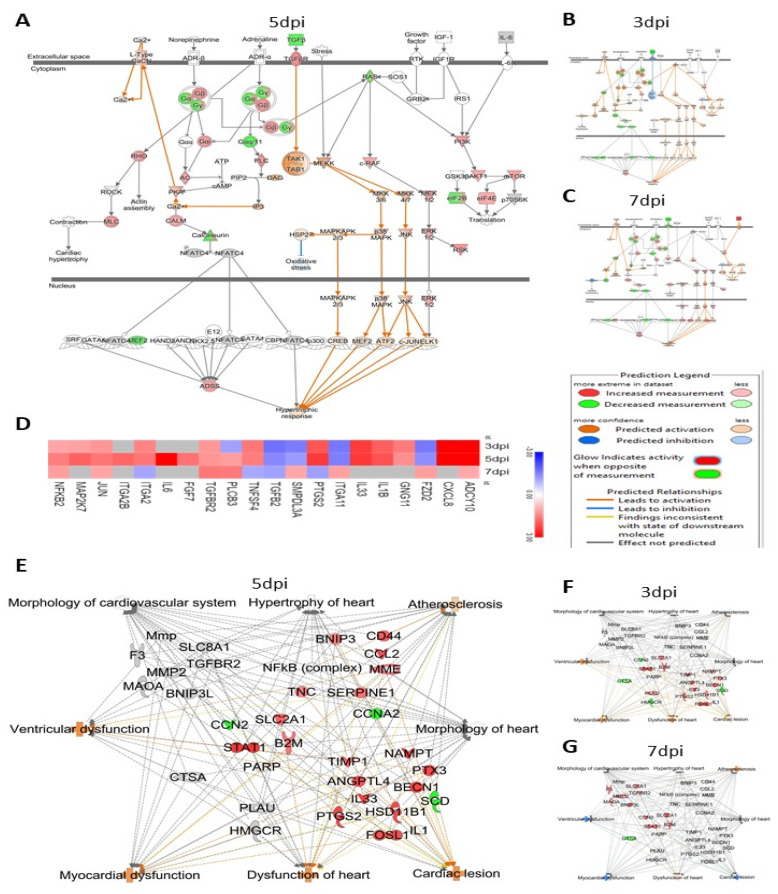
ZIKV infection significantly affects the levels of proteins involved in the cardiac hypertrophy pathway. IPA predicted activation of the cardiac hypertrophy pathway by Zika virus infection by (**A**) 3 dpi, (**B**) 7 dpi and (**C**) 5 dpi. (**D**). Heatmap of the proteins involved in cardiac hypertrophy pathway altered by ZIKV infection in HSerC. In the heatmap, red and blue represent up-regulation and down-regulation, respectively. Gray indicates the proteins were not detected at the respective time point. HSerC proteins associated with pathways that increase cardiovascular disease, that are affected by ZIKV infection at (**E**) 5 dpi (**F**) 3 dpi and (**G**) 7 dpi. Red and green represent up-regulation and down-regulation of protein expression, respectively. Abbreviations. dpi = Days post infection.

ZIKV infection significantly affected 20 proteins (Figure 8D) involved in the cardiac hypertrophy pathway and significantly activated (Z-score = 3.1) proteins within the pathway at 5 dpi (Figure 8A). However, at 3 dpi and 7 dpi, the proteins in this pathway were not significantly impacted by ZIKV infection (Figure 8B,C). IPA has a database of 617 proteins associated with the increase of cardiovascular disease. Among these, 23, 28 and 14 proteins were significantly affected by 3, 5 and 7 days post-ZIKV infection, respectively. The significantly altered proteins are associated with atherosclerosis, ventricular dysfunction, myocardial dysfunction, cardiac lesion, hypertrophy of heart, morphology of heart and cardiovascular system and dysfunction of heart (Figure 8E–G). 

### 3.7. Validation of Mass Spec Data by Western Blot

**Figure 9:**
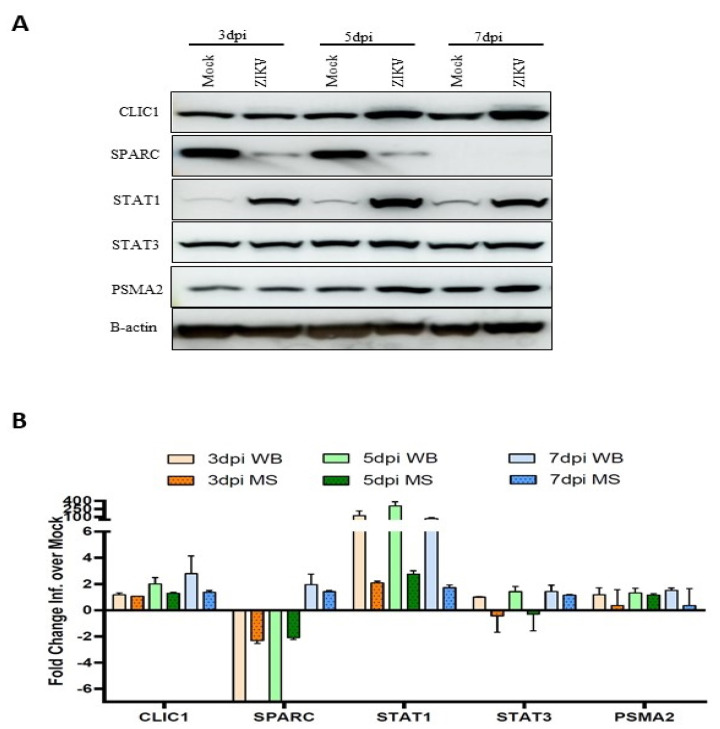
Western blot validation of proteomic changes detected by mass spectrometry (MS) analysis. Sertoli cell lysates were collected after ZIKV infection (MOI:3) at 3, 5 and 7 dpi. In total, 20 µg of proteins were separated by SDS-PAGE gel electrophoresis. (**A**) Expression of CLIC1, SPARC, STAT1, STAT3 and PSMA2 were detected by Western blot using specific antibodies. (**B**) Expression values of the proteins from Western-blot were quantified by ImageJ V1.8.0 from three replicates and plotted side-by-side to MS expression values. Expression of protein by Western blot was normalized to B-actin expression. Abbreviations: dpi = Days post infection; WB = Western blot; MS = Mass spectrometry; ZIKV = Zika virus.

Five proteins (CLIC1, SPARC, STAT1, STAT3 and PSMA2) were selected for validation based on their expression fold-change and availability of commercial antibodies. Western blot was done (Figure 9A), and the intensities of protein bands were measured to determine the fold difference between the infected and mock conditions. The fold change of each protein measured by Western blot at three different time points was compared side-by-side to the expression values determined by mass spectrometry (Figure 9B). All five proteins followed the same trend of expression by Western blot as measured by TMT-mass spectrometry.

## 4. Discussion

The mechanism(s) underlining the long-term persistence of ZIKV in male semen is not yet clearly understood. The sexual transmission of the virus raises the risk of initiating outbreaks in non-endemic regions, even in the absence of a mosquito vector. Furthermore, the pathologic consequences of viral persistence in the male genital tract are unclear. In our previous study, to investigate ZIKV infection-induced HSerC proteome alterations, we applied SOMAscan, a multi-plexed targeted technology that can identify over 1300 proteins from each sample [36]. Although SOMAscan, as a targeted approach, can identify many non-abundant proteins, it is still limited in its detection limit to the specified 1305 proteins [42]. Therefore, in this study, we used a six-plex TMT-based mass spectrometry approach to determine additional HSerC proteome alterations to provide a broader insight on the impact of ZIKV infection. However, unlike the previous study, we excluded the 1 dpi time point due to a small number of significantly affected proteins [36] and included 7 dpi to analyze the impact of ZIKV on Sertoli cells at a later stage. In addition, as previously reported [36,37,43], Zika virus infection had no apparent cytopathic impact on Sertoli cells after infection (Figure 1A). However, ZIKV replication [36] and viral protein expression (Figure 1B,C) were significantly reduced at 7 dpi in Sertoli cells, but infectious virus has been detected for at least 6 weeks in these cells [37]. This indicates that ZIKV initially undergoes a robust infection in Sertoli cells, but it is subsequently controlled, likely by cellular immune responses and later it maintains a low level of persistence. In this study, we have seen that most of the significantly affected proteins were down−regulated at 3 dpi, and up−regulated at 5 dpi as observed before [36]. This confirms a consistent time−dependent switch of proteomic alteration caused by ZIKV infection in Sertoli cells. Among the most affected proteins, FBXO11 has been associated with Neurodevelopmental disorders, mental retardation, and autism [44,45]. However, KIF1A expression anomaly was linked with neurological disorders in children [46]. Therefore, the alteration of FBXO11 and KIF1A could be associated with microcephaly and Guillain–Barré syndrome that develop during and/or after ZIKV infection. UHRF2 is one of the central regulators of cell cycle machinery [47]. TYR is responsible for initiating the conversion of tyrosine to melanin, which is responsible for pigmentation of eye, skin and hair [48]. Abnormalities in TYR expression may cause albinism [49,50]. FOXQ1 is a transcription regulator that controls the cell cycle, cell proliferation and is associated with cancers [51,52,53,54], and that inhibits macrophage recruitment [55], and activates Wnt signaling [56].

### 4.1. HSerC Activates Immune Response against ZIKV Infection

The human testis maintains an immune-privileged environment, separated from the body cavity by the blood–testis barrier (BTB), made with Sertoli cells [57]. However, to combat invading microbial pathogens, the testis possesses a local immune defense system [28]. In response to ZIKV infection, HSerC induced changes in the levels of proteins predicted to activate a strong immune response at 3, 5 and 7 dpi. According to the analysis of the 50 predominantly affected proteins, the inflammatory and antimicrobial response pathways were significantly triggered after ZIKV infection of HSerC (Figure 4D). The protein–protein interaction analysis also predicted antimicrobial response, infectious diseases, and inflammatory response as activated across the three-time points (Table S3). In addition, at least 17 cellular signaling pathways associated with immune response were predicted to be significantly activated in HSerC after ZIKV infection (Figure 5B). In agreement with our findings, a previous study based on transcriptomic analysis [35] also determined that interferon signaling, IL-15, role of PKR interferon induction and antiviral response, HMGB1 and role of pattern recognition receptor in recognition of bacteria and virus were significantly affected by ZIKV infection of Sertoli cells. Further analysis of the significantly affected proteins (Figure 5A) and upstream molecules (Figure S1B) indicated that proteins involved in immune response of antigen-presenting cell pathways (APCs), phagocytes, leukocytes and T lymphocytes were significantly activated. Sertoli cells’ potent immune response may have inhibited viral replication, as anticipated by IPA (Figure 6B), also seen by the decreased quantity of viral proteins at 5 and 7 dpi (Figure 1B,C). Interestingly, proteins associated with antiviral response against Flaviviridae may significantly suppress other human viruses such as Hepatitis C virus, Herpesvirus, and Coronavirus (Figure 6A,B). This suggests that targeting commonly used host proteins or pathways might lead to the development of a broad-spectrum antiviral therapy in the future.

### 4.2. ZIKV Affects Proteins Involved in Carbohydrate Metabolism 

Viruses use biomolecules from the host and induce anabolism to create the macromolecules needed for virion replication and assembly. As a result, it is no surprise that viral infection forces host cells to modify their metabolism in order to support effective virus replication [58]. Many oncogenic viruses use the glycolysis pathway, such as human papillomavirus (HPV), hepatitis C virus (HCV), hepatitis B virus (HBV), Kaposi’s sarcoma-associated herpesvirus (KSHV), Epstein–Barr virus (EBV), Merkel cell polyomavirus (MCPyV), and Adenovirus [59,60]. However, several non-oncogenic viruses were also found to activate the glycolysis pathways, including Herpes simplex virus 1 and 2 [61], and Human cytomegalovirus [58]. Dengue virus (DENV), another relative of ZIKV, depends on the glycolysis pathways for its replication [62]. High concentrations of glucose can restrict the growth of ZIKV in human kidney cells [63]. ZIKV infection increases glucose incorporation into the TCA cycle in mosquito cells, but the mechanism is not clear [64]. In HSerC, proteins involved in glycolysis and gluconeogenesis pathways were significantly activated by ZIKV infection (Figure 3A and Figure 7B). In addition, the metabolism of d-fructose, monosaccharides, phosphatidic acid, phospholipids were also predicted to be significantly activated by the significantly altered proteins (Figure 7C, Figure S3). Previously, we detected PGAM1, a key regulator of glycolysis and gluconeogenesis pathways, as significantly up-regulated by ZIKV infection in HSerC [36]. However, proteins involved in energy generation in Sertoli cells by lactate and lipid oxidization through the Peroxisome Proliferator-Activated Receptor (PPAR) signaling pathway [65] were significantly down-regulated by ZIKV infection (Figure 3A), as reported previously [36]. These results suggest that ZIKV hijacks carbohydrate metabolism to usurp cellular energy generation in HSerC. The glycolysis pathway could be a potential target against ZIKV and needs further investigation in the future. 

### 4.3. ZIKV Infection Affects Proteins Associated with an Increase in Cardiovascular Disease

Surprisingly, we found many proteins involved in cardiac disease that were significantly affected (Figure 8E–G) during ZIKV infection of HSerC. These proteins are associated with significant activation of atherosclerosis, ventricular dysfunction, myocardial dysfunction, and cardiac lesion (Figure 8E–G). RGS5 is one of the topmost affected proteins detected in HSerC after ZIKV infection. It is a GTPase activator that protects against cardiac hypertrophy and cardiac fibrosis [66]. Cardiovascular disease was a frequently detected protein–protein network predicted by IPA at all three−time points after ZIKV infection (Table S3). In addition, levels of at least 20 proteins involved in the cardiac hypertrophy signaling pathway were significantly altered by ZIKV, causing significant predicted activation of the pathway (Figure 3A and Figure 8A) at 5 dpi in HSerC. Thus far, cardiovascular complications have been associated with ZIKV infection by a case report [67], an observational study [68], and in an animal study [69]. Although identified by us in HSerC, the cardiac hypertrophy signaling pathway could be a possible mechanism of cardiac complications in ZIKV-infected patients. However, the current study has limitations, as it was performed in a non-cardiac cell line. To fully comprehend the link between ZIKV and cardiovascular abnormalities and its mechanism, extensive investigation is necessary.

## 5. Conclusions

Understanding the mechanisms of ZIKV persistence in the male genital tract is critical for treatment of the disease, antiviral/vaccine development and overall control of viral transmission. Multiple studies have suggested that Sertoli cells could be a potential reservoir for this viral persistence. Sertoli cells build the blood–testis barrier that maintains an isolated environment for optimal development of the germ cells and provides sufficient nutrients and signals for sequential differentiation into sperm by spermatogenesis [30]. ZIKV infection of Sertoli cells may impact sperm development and male fertility. Moreover, a clear understanding of the replication dynamics of the virus in an immune−privileged environment is necessary for antiviral or vaccine development. Previous studies of ZIKV−infected Sertoli cells have reported the roles of a particular protein [70] or its impact on the BTB [71]. However, this is the first study that uses mass spectrometry to understand global proteomic alterations induced by ZIKV infection in HSerC at 3, 5 and 7 dpi. Longitudinal proteome analysis facilitates the identification of critical proteins and pathways for the successful ZIKV infection cycle and provides detailed knowledge that may be used in therapeutic intervention. Many host proteins and signaling pathways crucial for ZIKV replication were found in this study, and these could be targeted for antiviral or diagnostic tool development in the future.

## Figures and Tables

**Table 1:** Numbers of significantly affected ZIKV-infected Sertoli proteins.

Number That Are Significant	Total Unique	3 dpi	5 dpi	7 dpi
and F.C. > 1.00	4423	853	1651	1209
and F.C. < 1.00		1514	712	573
and F.C. > 1.10	4361	832	1632	1197
and F.C. < −1.10		1495	700	559
and F.C. > 1.25	2234	393	744	647
and F.C. < −1.25		755	329	193
and F.C. > 1.33	1342	286	468	382
and F.C. < −1.33		395	197	90
and F.C. > 1.50	619	180	246	176
and F.C. < −1.50		140	97	22
and F.C. > 1.66	351	119	161	94
and F.C. < −1.66		71	45	7
and F.C. > 2.00	173	70	95	39
and F.C. < −2.00		24	14	3
and F.C. > 2.50	79	36	38	19
and F.C. < −2.50		6	8	1

Significance was determined by *t*-test and Z-score as detailed in Materials and Methods from three biological replicates. The 173 specific proteins whose levels were altered > 2.0-fold in either direction are listed in Table 2.

**Table 2:** List of protein levels altered at least 2-fold and significant by *t*-test (*p*-value < 0.05) or Z-score (≥1.96σ or ≤−1.96σ).

Swprot	Gene	Protein	3 dpi		5 dpi		7 dpi	
			Inf/Mock F.C.	*p*-Value	Inf/Mock F.C.	*p*-Value	Inf/Mock F.C.	*p*-Value
Up-regulated proteins								
O15539	RGS5	Regulator of G-protein signaling 5	45.46	5.5 × 10^−3^				
Q86XK2	FBXO11	F-box only protein 11	41.16	4.9 × 10^−3^	−1.01	7.9 × 10^−1^	1.11	1.9 × 10^−1^
Q96PU4	UHRF2	E3 ubiquitin-protein ligase UHRF2	17.55	1.1 × 10^−3^	5.74	2.4 × 10^−4^	−1.10	2.5 × 10^−1^
Q12955	ANK3	Ankyrin-3	13.21	8.1 × 10^−4^	3.36	6.5 × 10^−4^	2.29	3.8 × 10^−3^
Q8N6Y2	LRRC17	Leucine-rich repeat-containing protein 17	9.94	4.9 × 10^−4^	2.82	1.4 × 10^−4^	1.31	9.5 × 10^−3^
O15544	LINC01565	Protein GR6	5.71	1.8 × 10^−4^				
P14679	TYR	Tyrosinase	4.85	2.6 × 10^−4^	15.63	1.8 × 10^−3^		
O95294	RASAL1	RasGAP-activating-like protein 1	4.80	8.5 × 10^−4^	2.08	4.0 × 10^−2^		
Q9P275	USP36	Ubiquitin carboxyl-terminal hydrolase 36	4.44	1.2 × 10^−4^	1.03	7.0 × 10^−1^	1.06	3.5 × 10^−1^
Q96PN6	ADCY10	Adenylate cyclase type 10	4.39	8.8 × 10^−5^	4.19	2.8 × 10^−4^	1.00	9.1 × 10^−1^
P61371	ISL1	Insulin gene enhancer protein ISL-1	4.11	1.6 × 10^−4^				
P10145	CXCL8	Interleukin-8	3.83	1.8 × 10^−4^	3.07	4.5 × 10^−4^		
P20591	MX1	Interferon-induced GTP-binding protein Mx1	3.53	8.1 × 10^−5^	5.24	1.0 × 10^−4^	4.54	4.8 × 10^−4^
Q6ZV65	FAM47E	Protein FAM47E	3.53	4.9 × 10^−5^				
Q13438	OS9	Protein	3.42	2.1 × 10^−4^	0.99	8.0 × 10^−1^	1.04	5.7 × 10^−1^
Q8N9S9	SNX31	Sorting nexin-31	3.31	1.0 × 10^−4^				
O14879	IFIT3	Interferon-induced protein with tetratricopeptide repeats 3	3.30	3.5 × 10^−5^	3.41	9.5 × 10^−5^	3.75	4.2 × 10^−4^
Q8TEJ3	SH3RF3	SH3 domain-containing RING finger protein 3	3.22	2.4 × 10^−4^	1.01	8.1 × 10^−1^		
P30447	HLA-A	HLA class I histocompatibility antigen, A-23 alpha chain	3.17	1.8 × 10^−4^	3.96	1.5 × 10^−4^		
Q96EN8	MOCOS	Molybdenum cofactor sulfurase	3.12	1.4 × 10^−4^	2.20	6.7 × 10^−4^	2.23	3.4 × 10^−3^
Q9BXS9	SLC26A6	Solute carrier family 26 member 6	3.10	7.0 × 10^−5^	1.21	1.1 × 10^−2^	1.13	1.5 × 10^−1^
P29728	OAS2	2’-5’-oligoadenylate synthase 2	2.98	1.9 × 10^−4^	3.54	1.8 × 10^−4^	3.28	9.1 × 10^−4^
Q9C002	NMES1	Normal mucosa of esophagus-specific gene 1 protein	2.96	1.7 × 10^−4^	2.91	1.4 × 10^−4^	1.18	1.3 × 10^−1^
P05161	ISG15	Ubiquitin-like protein ISG15	2.92	5.9 × 10^−5^	2.20	4.3 × 10^−5^	2.93	2.1 × 10^−4^
P02774	GC	Vitamin D-binding protein	2.87	4.5 × 10^−5^	1.86	3.8 × 10^−4^	1.34	4.1 × 10^−5^
Q9Y6K5	OAS3	2’-5’-oligoadenylate synthase 3	2.85	4.3 × 10^−5^	3.60	1.1 × 10^−4^	2.30	1.4 × 10^−3^
P09913	IFIT2	Interferon-induced protein with tetratricopeptide repeats 2	2.82	6.5 × 10^−5^	2.83	5.7 × 10^−5^	2.83	8.4 × 10^−4^
Q14207	NPAT	Protein NPAT	2.78	3.3 × 10^−4^				
P09914	IFIT1	Interferon-induced protein with tetratricopeptide repeats 1	2.76	2.4 × 10^−5^	3.05	8.4 × 10^−5^	2.76	9.0 × 10^−4^
Q06055	ATP5G2	ATP synthase F(0) complex subunit C2, mitochondrial	2.73	2.8 × 10^−5^				
Q8TCB0	IFI44	Interferon-induced protein 44	2.68	1.1 × 10^−4^	2.07	4.5 × 10^−4^	2.37	7.5 × 10^−4^
O75071	EFCAB14	EF-hand calcium-binding domain-containing protein 14	2.67	1.7 × 10^−3^	1.06	2.9 × 10^−1^		
Q8N2C7	UNC80	Protein unc-80 homolog	2.66	1.6 × 10^−4^				
Q92901	RPL3L	60S ribosomal protein L3-like	2.65	2.4 × 10^−3^			1.97	1.6 × 10^−2^
Q92766	RREB1	Ras-responsive element-binding protein 1	2.56	7.7 × 10^−4^	3.72	2.1 × 10^−4^		
Q96LM1	LINC00615	Putative uncharacterized protein encoded by LINC00615	2.56	3.0 × 10^−3^				
P47895	ALDH1A3	Aldehyde dehydrogenase family 1 member A3	2.50	3.4 × 10^−5^	3.86	9.7 × 10^−5^	1.38	2.9 × 10^−2^
P20592	MX2	Interferon-induced GTP-binding protein Mx2	2.48	9.3 × 10^−5^	2.98	1.2 × 10^−4^	2.80	4.1 × 10^−4^
Q96AZ6	ISG20	Interferon-stimulated gene 20 kDa protein	2.47	3.0 × 10^−5^	2.46	3.0 × 10^−4^		
Q9BXU1	STK31	Serine/threonine-protein kinase 31	2.47	3.2 × 10^−4^				
Q9Y3Z3	SAMHD1	Deoxynucleoside triphosphate triphosphohydrolase SAMHD1	2.46	1.0 × 10^−4^	2.82	4.5 × 10^−5^	2.49	1.1 × 10^−3^
P52823	STC1	Stanniocalcin-1	2.43	2.6 × 10^−4^	2.98	1.4 × 10^−4^		
P02795	MT2A	Metallothionein-2	2.42	8.0 × 10^−6^	6.81	6.8 × 10^−4^		
P30464	HLA-B	HLA class I histocompatibility antigen, B-15 alpha chain	2.41	2.3 × 10^−4^	2.49	1.6 × 10^−4^	2.11	1.5 × 10^−3^
A6ND36	FAM83G	Protein FAM83G	2.33	1.6 × 10^−4^	1.77	1.9 × 10^−4^	1.14	7.0 × 10^−4^
P48307	TFPI2	Tissue factor pathway inhibitor 2	2.32	1.4 × 10^−4^	3.11	1.9 × 10^−4^	1.18	4.1 × 10^−2^
O95760	IL33	Interleukin-33	2.30	4.4 × 10^−4^	2.35	1.1 × 10^−4^		
Q6UXH9	PAMR1	Inactive serine protease PAMR1	2.26	2.1 × 10^−4^	1.12	7.5 × 10^−2^		
P09912	IFI6	Interferon alpha-inducible protein 6	2.23	5.0 × 10^−4^	2.87	2.2 × 10^−4^		
Q71F56	MED13L	Mediator of RNA polymerase II transcription subunit 13-like	2.23	3.2 × 10^−4^			1.04	8.1 × 10^−1^
Q8TDJ6	DMXL2	DmX-like protein 2	2.21	1.1 × 10^−3^	3.02	1.9 × 10^−4^		
Q6P589	TNFAIP8L2	Tumor necrosis factor alpha-induced protein 8-like protein 2	2.20	1.9 × 10^−4^	2.59	6.9 × 10^−4^	−1.13	2.2 × 10^−1^
P18464	HLA-B	HLA class I histocompatibility antigen, B-51 alpha chain	2.18	7.6 × 10^−4^	2.78	1.5 × 10^−4^	2.62	9.1 × 10^−4^
Q10589	BST2	Bone marrow stromal antigen 2	2.14	3.7 × 10^−5^	3.95	2.1 × 10^−4^	3.47	3.5 × 10^−4^
P01033	TIMP1	Metalloproteinase inhibitor 1	2.11	4.2 × 10^−5^	2.02	4.5 × 10^−4^	1.11	1.3 × 10^−1^
Q96J88	EPSTI1	Epithelial-stromal interaction protein 1	2.10	8.8 × 10^−4^			1.57	7.8 × 10^−3^
O14933	UBE2L6	Ubiquitin/ISG15-conjugating enzyme E2 L6	2.09	2.2 × 10^−5^	2.71	2.5 × 10^−4^	1.73	7.4 × 10^−3^
P30685	HLA-B	HLA class I histocompatibility antigen, B-35 alpha chain	2.09	1.3 × 10^−4^	2.28	2.8 × 10^−4^	2.00	2.0 × 10^−3^
Q86UQ4	ABCA13	ATP-binding cassette sub-family A member 13	2.09	1.9 × 10^−3^				
P42224	STAT1	Signal transducer and activator of transcription 1-alpha/beta	2.08	1.4 × 10^−5^	2.74	5.8 × 10^−5^	1.71	3.3 × 10^−3^
P15407	FOSL1	Fos-related antigen 1	2.08	1.5 × 10^−4^	2.18	3.2 × 10^−4^		
O14791	APOL1	Apolipoprotein L1	2.07	5.5 × 10^−4^				
Q9UII4	HERC5	E3 ISG15--protein ligase HERC5	2.07	5.5 × 10^−4^	3.23	5.5 × 10^−4^	1.75	3.4 × 10^−3^
O00182	LGALS9	Galectin-9	2.07	1.8 × 10^−4^	2.69	1.2 × 10^−4^	1.67	3.9 × 10^−3^
Q14520	HABP2	Hyaluronan-binding protein 2	2.06	6.7 × 10^−4^	1.65	2.4 × 10^−4^	1.38	6.3 × 10^−3^
Q8IXQ6	PARP9	Poly [ADP-ribose] polymerase 9	2.05	2.8 × 10^−5^	2.11	3.3 × 10^−4^	1.77	1.6 × 10^−3^
Q03405	PLAUR	Urokinase plasminogen activator surface receptor	2.05	5.0 × 10^−5^	1.53	2.0 × 10^−3^	1.41	7.8 × 10^−4^
Q96L93	KIF16B	Kinesin-like protein KIF16B	2.04	2.1 × 10^−3^			1.13	1.4 × 10^−1^
Q29960	HLA-C	HLA class I histocompatibility antigen, Cw-16 alpha chain	2.04	2.1 × 10^−4^	2.37	4.4 × 10^−4^		
P25774	CTSS	Cathepsin S	2.02	4.8 × 10^−4^	1.79	3.3 × 10^−4^		
P04733	MT1F	Metallothionein-1F			3.99	5.9 × 10^−4^		
Q9Y5P4	COL4A3BP	Collagen type IV alpha-3-binding protein	1.08	1.3 × 10^−1^	3.52	2.6 × 10^−4^	−1.03	5.5 × 10^−1^
P05231	IL6	Interleukin-6			3.51	1.1 × 10^−4^		
P21589	NT5E	5’-nucleotidase	1.98	8.2 × 10^−5^	2.87	1.2 × 10^−4^	1.88	1.6 × 10^−3^
P09341	CXCL1	Growth-regulated alpha protein			2.76	6.6 × 10^−4^		
Q8N8U9	BMPER	BMP-binding endothelial regulator protein			2.62	7.6 × 10^−5^		
O15162	PLSCR1	Phospholipid scramblase 1	1.84	9.3 × 10^−4^	2.61	5.0 × 10^−4^	2.81	6.2 × 10^−4^
P43490	NAMPT	Nicotinamide phosphoribosyltransferase	1.55	3.6 × 10^−5^	2.60	1.5 × 10^−4^	−1.01	8.9 × 10^−1^
P02549	SPTA1	Spectrin alpha chain, erythrocytic 1	1.16	2.8 × 10^−2^	2.49	8.1 × 10^−6^		
Q9H5V8	CDCP1	CUB domain-containing protein 1	1.87	2.7 × 10^−4^	2.47	1.9 × 10^−4^	1.44	7.9 × 10^−4^
P04179	SOD2	Superoxide dismutase [Mn], mitochondrial	1.54	1.2 × 10^−3^	2.47	8.6 × 10^−5^	−1.27	2.2 × 10^−2^
O43581	SYT7	Synaptotagmin-7			2.45	7.5 × 10^−3^		
Q8NAP3	ZBTB38	Zinc finger and BTB domain-containing protein 38	1.15	6.6 × 10^−3^	2.44	4.6 × 10^−4^	1.06	3.6 × 10^−1^
Q53G44	IFI44L	Interferon-induced protein 44-like	1.83	3.3 × 10^−4^	2.34	1.5 × 10^−4^	2.38	5.3 × 10^−4^
Q14457	BECN1	Beclin-1	1.77	1.1 × 10^−4^	2.32	6.7 × 10^−5^	1.03	5.9 × 10^−1^
P35354	PTGS2	Prostaglandin G/H synthase 2	1.99	3.7 × 10^−4^	2.31	1.5 × 10^−5^	−1.13	2.9 × 10^−1^
Q8WZ74	CTTNBP2	Cortactin-binding protein 2			2.27	5.4 × 10^−4^	1.24	1.9 × 10^−2^
P26022	PTX3	Pentraxin-related protein PTX3	1.54	3.3 × 10^−4^	2.26	2.5 × 10^−3^	−1.05	5.1 × 10^−1^
Q5EBM0	CMPK2	UMP-CMP kinase 2, mitochondrial	1.91	1.1 × 10^−4^	2.25	9.5 × 10^−5^	1.84	1.3 × 10^−3^
P28845	HSD11B1	Corticosteroid 11-beta-dehydrogenase isozyme 1	1.56	1.6 × 10^−3^	2.23	2.6 × 10^−4^	1.19	3.7 × 10^−2^
Q8WWZ7	ABCA5	ATP-binding cassette sub-family A member 5			2.23	5.5 × 10^−5^	1.13	1.2 × 10^−1^
O95786	DDX58	Probable ATP-dependent RNA helicase DDX58	1.79	1.2 × 10^−4^	2.23	2.1 × 10^−4^	2.11	1.3 × 10^−3^
P04732	MT1E	Metallothionein-1E	1.26	3.8 × 10^−3^	2.22	1.5 × 10^−4^	1.91	2.3 × 10^−3^
Q96RQ9	IL4I1	l-amino-acid oxidase	1.74	3.3 × 10^−4^	2.22	1.6 × 10^−4^	1.28	2.8 × 10^−2^
P01892	HLA-A	HLA class I histocompatibility antigen, A-2 alpha chain	1.78	2.8 × 10^−4^	2.22	4.1 × 10^−4^	1.60	4.9 × 10^−3^
Q4VCS5	AMOT	Angiomotin	1.92	4.8 × 10^−4^	2.20	1.1 × 10^−4^		
P00973	OAS1	2’-5’-oligoadenylate synthase 1	1.99	1.0 × 10^−4^	2.20	1.3 × 10^−3^	2.02	3.5 × 10^−3^
Q5TEJ8	THEMIS2	Protein THEMIS2			2.18	6.1 × 10^−5^		
Q96C10	DHX58	Probable ATP-dependent RNA helicase DHX58	1.35	1.6 × 10^−2^	2.18	1.8 × 10^−4^	1.08	2.6 × 10^−1^
P13500	CCL2	C-C motif chemokine 2			2.17	7.9 × 10^−4^		
Q9BY76	ANGPTL4	Angiopoietin-related protein 4	1.58	4.7 × 10^−4^	2.17	8.3 × 10^−4^	1.36	1.0 × 10^−2^
Q9Y6I4	USP3	Ubiquitin carboxyl-terminal hydrolase 3	1.01	7.1 × 10^−1^	2.16	1.3 × 10^−4^		
O94808	GFPT2	Glutamine--fructose-6-phosphate aminotransferase [isomerizing] 2	1.65	6.4 × 10^−5^	2.15	2.6 × 10^−4^	1.38	6.8 × 10^−3^
P15153	RAC2	Ras-related C3 botulinum toxin substrate 2	1.58	2.7 × 10^−4^	2.14	5.2 × 10^−5^	1.04	3.8 × 10^−1^
P19525	EIF2AK2	Interferon-induced, double-stranded RNA-activated protein kinase	1.59	1.1 × 10^−4^	2.13	1.1 × 10^−4^	1.91	1.6 × 10^−3^
Q9HB58	SP110	Sp110 nuclear body protein	1.80	1.7 × 10^−3^	2.11	9.3 × 10^−4^	1.81	1.2 × 10^−3^
P05534	HLA-A	HLA class I histocompatibility antigen, A-24 alpha chain	1.58	4.0 × 10^−4^	2.09	1.9 × 10^−4^	1.98	8.2 × 10^−4^
P52926	HMGA2	High mobility group protein HMGI-C	1.28	2.5 × 10^−2^	2.09	2.2 × 10^−5^	−1.16	5.7 × 10^−2^
O75508	CLDN11	Claudin-11	1.05	5.9 × 10^−1^	2.09	3.9 × 10^−4^	1.27	2.1 × 10^−3^
P07148	FABP1	Fatty acid-binding protein, liver			2.09	4.4 × 10^−4^	−1.09	4.6 × 10^−1^
Q7Z402	TMC7	Transmembrane channel-like protein 7			2.08	2.3 × 10^−4^		
Q01973	ROR1	Inactive tyrosine-protein kinase transmembrane receptor ROR1			2.08	4.6 × 10^−4^	1.41	2.1 × 10^−3^
Q8IVT2	MISP	Mitotic interactor and substrate of PLK1	1.75	3.8 × 10^−4^	2.07	4.0 × 10^−4^		
Q3MIT2	PUS10	Putative tRNA pseudouridine synthase Pus10			2.06	4.3 × 10^−3^	−1.31	5.5 × 10^−3^
P27701	CD82	CD82 antigen	1.34	1.6 × 10^−2^	2.05	1.5 × 10^−5^	1.22	4.3 × 10^−2^
Q15646	OASL	2’-5’-oligoadenylate synthase-like protein	1.82	1.3 × 10^−4^	2.05	2.8 × 10^−4^	1.99	1.8 × 10^−3^
P01584	IL1B	Interleukin-1 beta	1.88	9.0 × 10^−5^	2.04	2.1 × 10^−4^		
Q7Z3S9	NOTCH2NL	Notch homolog 2 N-terminal-like protein	1.13	1.0 × 10^−1^	2.02	1.7 × 10^−4^	1.03	6.1 × 10^−1^
P68871	HBB	Hemoglobin subunit beta	1.54	3.6 × 10^−3^	2.02	2.4 × 10^−4^	1.31	1.3 × 10^−2^
Q9BYX4	IFIH1	Interferon-induced helicase C domain-containing protein 1	1.82	1.8 × 10^−4^	2.01	2.5 × 10^−4^	2.09	2.7 × 10^−3^
P16070	CD44	CD44 antigen	1.42	8.9 × 10^−4^	2.01	2.2 × 10^−4^	1.32	3.6 × 10^−3^
P28838	LAP3	Cytosol aminopeptidase	1.66	2.3 × 10^−5^	2.00	1.4 × 10^−4^	1.42	6.2 × 10^−3^
Q92597	NDRG1	Protein NDRG1	1.21	1.6 × 10^−3^	2.00	1.4 × 10^−4^	1.75	7.5 × 10^−4^
P11166	SLC2A1	Solute carrier family 2, facilitated glucose transporter member 1	1.65	6.2 × 10^−4^	1.91	7.9 × 10^−4^	2.12	9.2 × 10^−6^
P09871	C1S	Complement C1s subcomponent	1.08	2.3 × 10^−1^	1.25	5.7 × 10^−3^	2.10	1.3 × 10^−3^
P11169	SLC2A3	Solute carrier family 2, facilitated glucose transporter member 3	1.28	3.2 × 10^−3^	1.21	2.2 × 10^−2^	2.71	2.3 × 10^−5^
Q96DE5	ANAPC16	Anaphase-promoting complex subunit 16	−1.12	2.0 × 10^−1^	1.13	8.3 × 10^−3^	3.57	4.4E−10
P00966	ASS1	Argininosuccinate synthase	−1.12	1.1 × 10^−1^	−1.13	2.1 × 10^−2^	2.08	1.6 × 10^−3^
Q2UY09	COL28A1	Collagen alpha-1(XXVIII) chain	−1.93	1.2 × 10^−3^	−1.71	3.1 × 10^−3^	2.95	3.6 × 10^−2^
P04264	KRT1	Keratin, type II cytoskeletal 1	−1.40	1.9 × 10^−1^	−1.98	1.9 × 10^−2^	−2.41	2.5 × 10^−6^
Q9HCJ2	LRRC4C	Leucine-rich repeat-containing protein 4C					7.23	1.3 × 10^−3^
Q8IXR9	C12orf56	Uncharacterized protein C12orf56					3.90	8.3 × 10^−3^
O15068	MCF2L	Guanine nucleotide exchange factor DBS	−1.93	1.6 × 10^−3^			3.47	3.1 × 10^−2^
Q9NQ90	ANO2	Anoctamin-2	1.23	2.1 × 10^−2^			2.86	1.2 × 10^−3^
P17693	HLA-G	HLA class I histocompatibility antigen, alpha chain G					2.59	1.9 × 10^−4^
Q8IZ26	ZNF34	Zinc finger protein 34					2.51	2.2 × 10^−3^
Q9Y225	RNF24	RING finger protein 24			1.13	5.7 × 10^−2^	2.42	8.5 × 10^−4^
P04222	HLA-C	HLA class I histocompatibility antigen, Cw-3 alpha chain					2.18	1.6 × 10^−4^
Q13794	PMAIP1	Phorbol-12-myristate-13-acetate-induced protein 1					2.12	3.1 × 10^−4^
Q96B67	ARRDC3	Arrestin domain-containing protein 3					2.09	2.3 × 10^−4^
Q13772	NCOA4	Nuclear receptor coactivator 4			−1.04	6.0 × 10^−1^	2.08	1.3 × 10^−3^
Q13137	CALCOCO2	Calcium-binding and coiled-coil domain-containing protein 2	1.19	3.4 × 10^−2^	−1.04	4.1 × 10^−1^	2.03	7.2 × 10^−4^
Down-regulated proteins								
Q12756	KIF1A	Kinesin-like protein KIF1A	−4.38	5.4 × 10^−5^				
P02452	COL1A1	Collagen alpha-1(I) chain	−3.95	1.5 × 10^−4^	−3.39	2.6 × 10^−4^	2.19	2.7 × 10^−2^
Q96K58	ZNF668	Zinc finger protein 668	−3.66	6.0 × 10^−5^	−2.63	1.6 × 10^−4^	1.03	3.6 × 10^−1^
P08123	COL1A2	Collagen alpha-2(I) chain	−3.39	1.0 × 10^−4^	−3.15	2.2 × 10^−4^	1.97	3.7 × 10^−2^
Q8IWF6	DENND6A	Protein DENND6A	−3.20	6.3 × 10^−4^				
P02461	COL3A1	Collagen alpha-1(III) chain	−2.87	3.2 × 10^−4^	−2.97	2.2 × 10^−4^	1.33	1.1 × 10^−2^
Q9H1P3	OSBPL2	Oxysterol-binding protein-related protein 2	−2.47	3.1 × 10^−4^	1.41	4.9 × 10^−3^		
Q8IZX4	TAF1L	Transcription initiation factor TFIID subunit 1-like	−2.46	5.1 × 10^−4^			−1.69	3.8 × 10^−3^
Q69YL0	NCBP2-AS2	Uncharacterized protein NCBP2-AS2	−2.42	6.9 × 10^−4^	−1.51	3.6 × 10^−3^	1.66	2.3 × 10^−2^
Q96JG9	ZNF469	Zinc finger protein 469	−2.41	5.0 × 10^−5^			1.03	7.2 × 10^−1^
P52732	KIF11	Kinesin-like protein KIF11	−2.34	6.7 × 10^−4^				
P09486	SPARC	SPARC	−2.32	3.7 × 10^−4^	−2.08	9.4 × 10^−4^	1.41	9.8 × 10^−3^
P20908	COL5A1	Collagen alpha-1(V) chain	−2.29	2.4 × 10^−4^	−1.99	4.9 × 10^−4^	1.40	4.5 × 10^−2^
Q96PQ7	KLHL5	Kelch-like protein 5	−2.20	3.6 × 10^−4^	−1.11	1.2 × 10^−1^		
P51911	CNN1	Calponin-1	−2.15	7.1 × 10^−4^	−2.51	1.7 × 10^−4^	1.12	2.0 × 10^−1^
Q8N7X1	RBMXL3	RNA-binding motif protein, X-linked-like-3	−2.13	5.0 × 10^−3^	−2.22	4.0 × 10^−3^		
O00767	SCD	Acyl-CoA desaturase	−2.13	4.9 × 10^−4^	−1.69	2.1 × 10^−3^	1.31	5.7 × 10^−3^
P61916	NPC2	Epididymal secretory protein E1	−2.10	4.3 × 10^−4^	−2.09	3.1 × 10^−4^	−1.52	4.1 × 10^−3^
Q05682	CALD1	Caldesmon	−2.08	2.7 × 10^−4^	−1.95	6.8 × 10^−4^	−1.15	1.1 × 10^−1^
Q8N806	UBR7	Putative E3 ubiquitin-protein ligase UBR7	−2.06	1.3 × 10^−3^	1.47	2.6 × 10^−3^	1.03	6.3 × 10^−1^
Q15113	PCOLCE	Procollagen C-endopeptidase enhancer 1	−2.06	4.6 × 10^−4^	−1.65	7.8 × 10^−4^	1.39	1.7 × 10^−2^
O75094	SLIT3	Slit homolog 3 protein	−2.04	3.5 × 10^−4^				
Q07352	ZFP36L1	Zinc finger protein 36, C3H1 type-like 1	−2.00	1.4 × 10^−4^	−1.03	5.6 × 10^−1^	1.42	5.6 × 10^−4^
P52736	ZNF133	Zinc finger protein 133	−2.00	1.7 × 10^−3^				
Q9C009	FOXQ1	Forkhead box protein Q1			−4.75	4.6 × 10^−5^		
Q96RY5	CRAMP1	Protein cramped-like			−4.03	1.1 × 10^−3^	1.01	7.7 × 10^−1^
P17661	DES	Desmin	−1.49	5.7 × 10^−3^	−2.63	1.8 × 10^−4^	1.39	4.5 × 10^−2^
P35527	KRT9	Keratin, type I cytoskeletal 9	−1.55	1.3 × 10^−1^	−2.35	4.0 × 10^−3^	−2.17	3.4 × 10^−5^
Q9NRM1	ENAM	Enamelin	−1.59	3.2 × 10^−4^	−2.31	2.8 × 10^−4^	1.51	3.1 × 10^−3^
O43854	EDIL3	EGF-like repeat and discoidin I-like domain-containing protein 3	−1.70	1.1 × 10^−3^	−2.16	8.2 × 10^−4^	−1.33	2.6 × 10^−3^
Q86YZ3	HRNR	Hornerin					−3.94	1.8 × 10^−13^

dpi = days post infection. FC = fold change, red = significantly up-regulated; green = significantly down-regulated, blue = *p*-value < 0.05. Table is sorted first by significantly up-regulated and downregulated proteins at 3 dpi, then by those significantly down-regulated at 5 dpi; then by significantly up- and down-regulated at 7 dpi.

## Data Availability

Spectra (in MGF format) and an overall log2 protein expression matrix are available at the University of California, San Diego’s MassIVE archive (massive.ucsd.edu) under the accession MSV000088634.

## Supplementary Materials

The following supporting information can be downloaded at: https://www.mdpi.com/article/10.3390/v14020377/s1, Supplementary Table S1. Disease and functions affected by ZIKV infection in HSerC. Supplementary Table S2. IPA predicted the upstream regulators affected by ZIKV infection in Sertoli cells. Supplementary Table S3. The protein–protein networks affected by ZIKV infection at 3,5 and 7 dpi in HSerC. Supplementary Figure S1. (A) Comparison of canonical pathways affected at 3, 5 and 7 dpi during ZIKV infection in HSerC. Red indicates up-regulation, blue indicates down-regulation. Numbers in the boxes show the significance of alteration measured by Z-score. (B) Top 15 upstream regulators associated with cellular function and disease. Supplementary Figure S2. IPA-predicted activation and inhibition of bio-functions, and protein–protein network at 3 and 7 dpi in HSerC. Top bio-functions and their predicted activation or inhibition Z-scores indicated at (A) 3 and (B) 7 dpi. Activation is indicated by positive Z-Score and the inhibited bio-functions are indicated by negative Z-scores. The most effected protein–protein networks at (C) 3 dpi and (D) 7 dpi by ZIKV infection in HSerC. Red and green represent up-regulation and down-regulation, respectively; gray proteins denote that they were recognized in the present study, but not significantly regulated; colorless proteins interact with molecules in the network, but were not identified in our study. Abbreviations: dpi = Days post infection. Supplementary Figure S3: Proteins involved in the metabolism of carbohydrate and energy production in cells affected by ZIKV infection at 3 dpi and 7 dpi. Red and green represent up-regulation and down-regulation, respectively; gray proteins denote that they were recognized in the present study, but not significantly regulated; colorless proteins interact with molecules in the network, but were not identified in our study. Abbreviations: dpi = Days post infection.
