# Supplementary material for: Zika Virus Infection of Sertoli Cells Alters Protein Expression Involved in Activated Immune and Antiviral Response Pathways, Carbohydrate Metabolism and Cardiovascular Disease
Source: Viruses. 2022 Feb 11;14(2):377. doi: 10.3390/v14020377 (PMC8878972; doi:10.3390/v14020377)
Supplement: Supplementary file 1 [file viruses-14-00377-s001.zip › viruses-1572177-suppl.pdf]

viruses

MDPI

Supplementary Materials

Zika Virus Infection of Sertoli Cells Alters Protein Expression Involved in Activated Immune and Antiviral Response Pathways, Carbohydrate Metabolism and Cardiovascular Disease

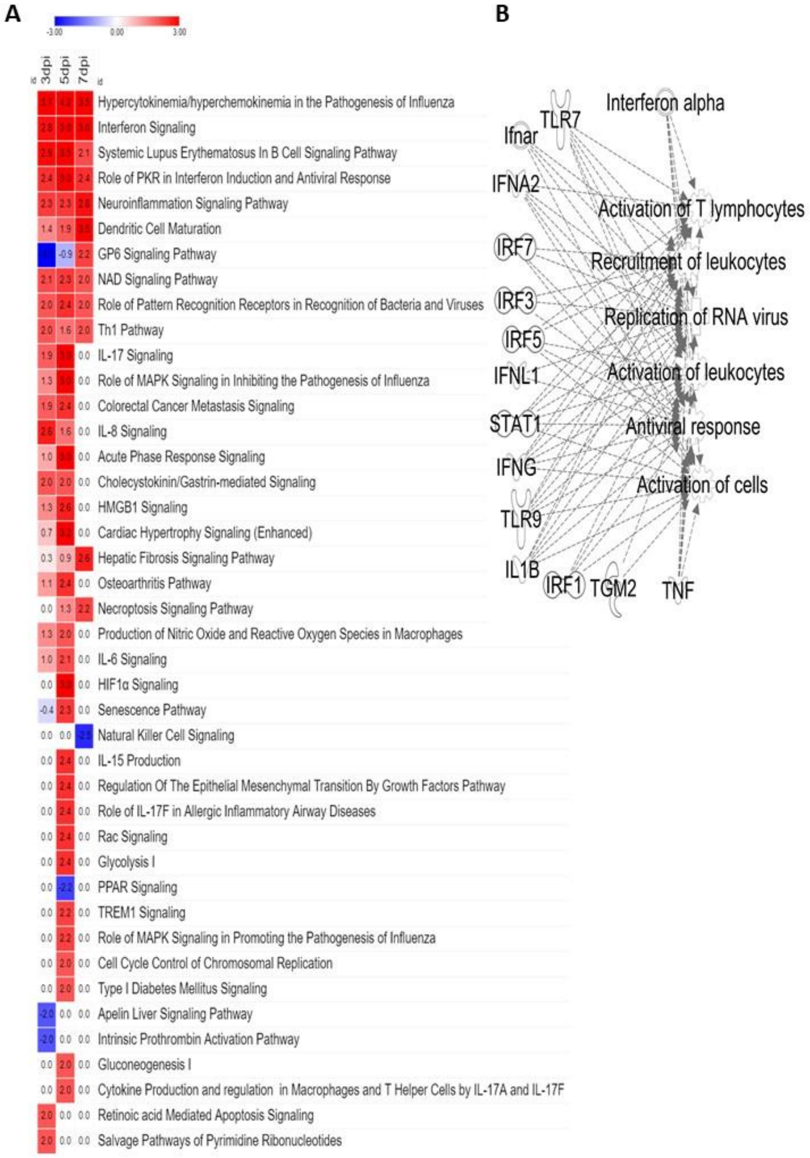

Figure S1. (**A**) Comparison of canonical pathways affected at 3, 5 and 7 dpi during ZIKV infection in HSerC. Red indicates up-regulation, blue indicates down-regulation. Numbers in the boxes show the significance of alteration measured by Z-score. (**B**) Top 15 upstream regulators associated with cellular function and disease.

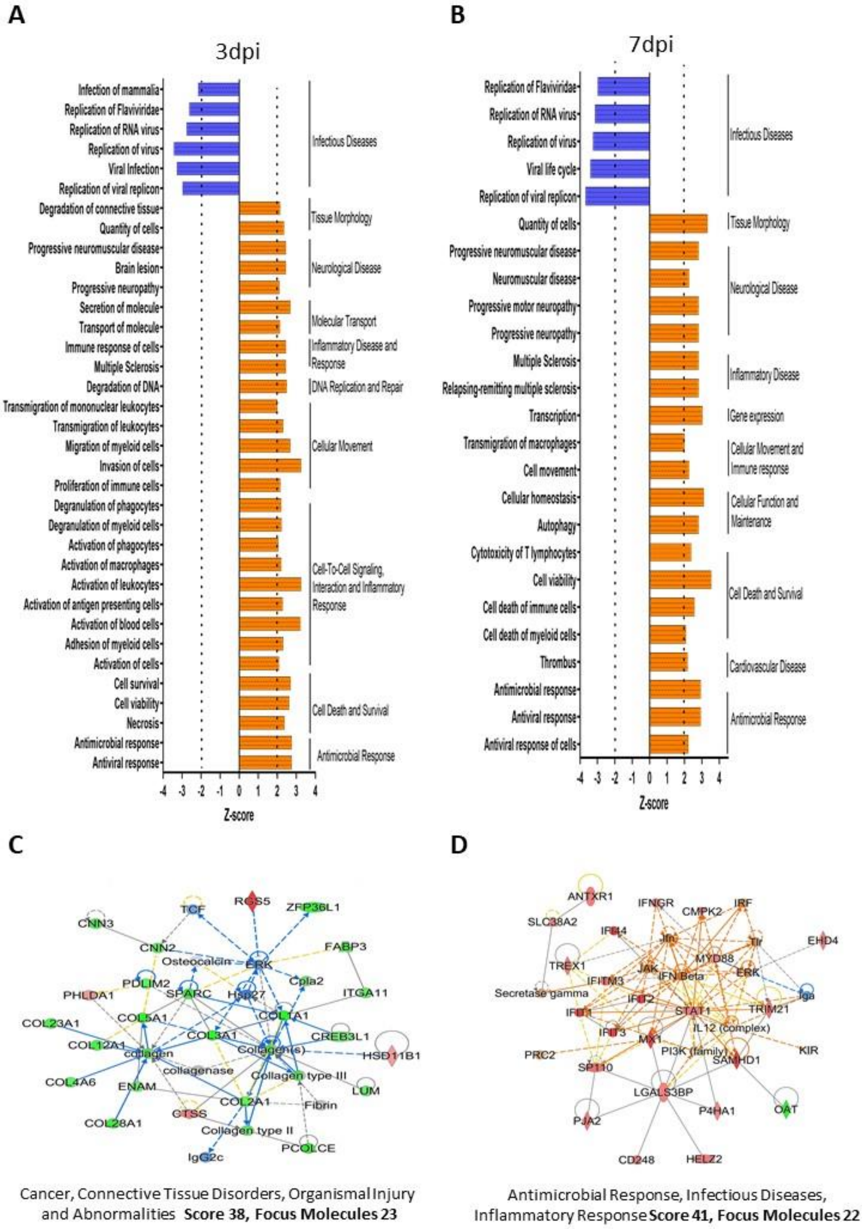

Figure S2. IPA-predicted activation and inhibition of bio-functions, and protein–protein network at 3 and 7 dpi in HSerC. Top bio-functions and their predicted activation or inhibition Z-scores indicated at (**A**) 3 and (**B**) 7 dpi. Activation is indicated by positive Z-Score and the inhibited bio-functions are indicated by negative Z-scores. The most effected protein–protein networks at (**C**) 3 dpi and (**D**) 7 dpi by ZIKV infection in HSerC. Red and green represent up-regulation and down-regulation, respectively; gray proteins denote that they were recognized in the present study, but not significantly regulated; colorless proteins interact with molecules in the network, but were not identified in our study. Abbreviations: dpi = Days post infection.

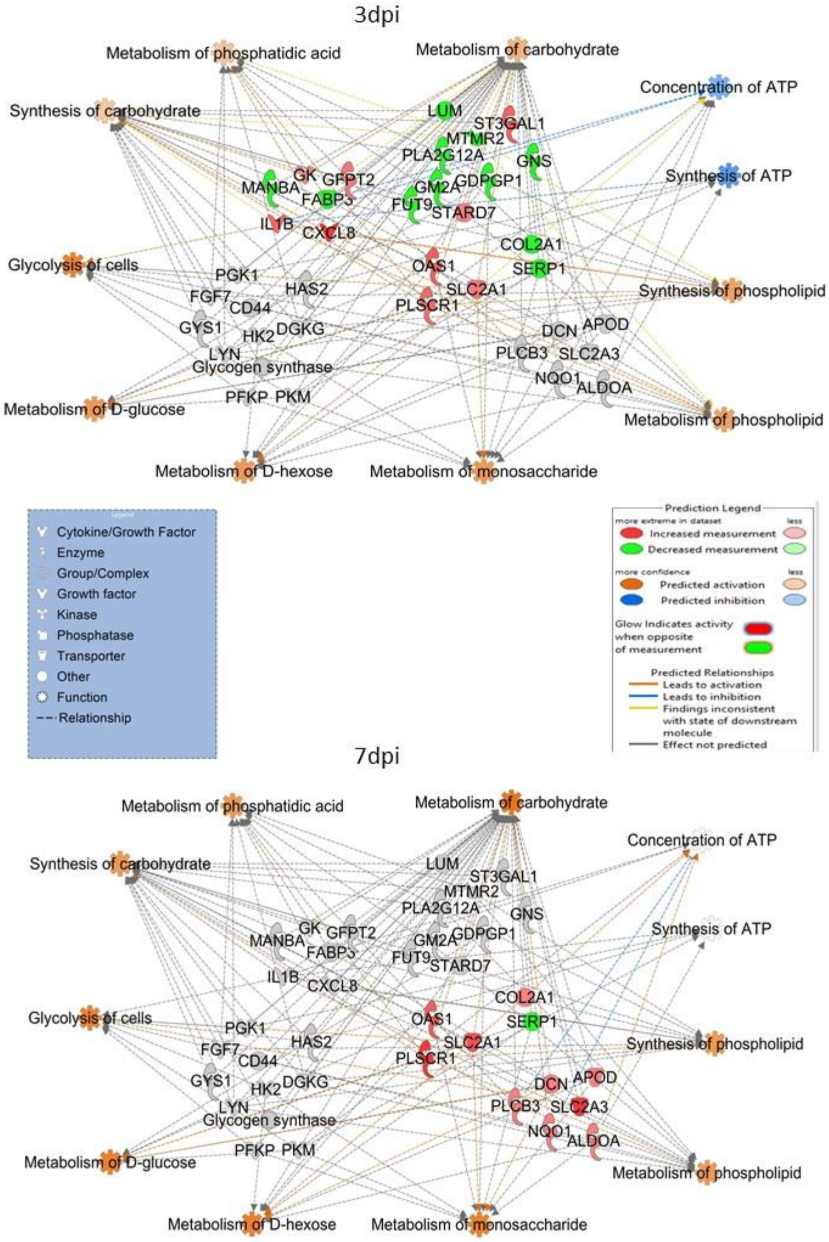

Figure S3. Proteins involved in the metabolism of carbohydrate and energy production in cells affected by ZIKV infection at 3 dpi and 7 dpi. Red and green represent up-regulation and down-regulation, respectively; gray proteins denote that they were recognized in the present study, but not significantly regulated; colorless proteins interact with molecules in the network, but were not identified in our study. Abbreviations: dpi = Days post infection.
